# Examination of Cutaneous Changes Among Patients Following SARS-CoV-2 Infection

**DOI:** 10.7759/cureus.27052

**Published:** 2022-07-20

**Authors:** Jordan B Lane, Samuel Stahly, Adam Sills, Stephen D Wagner, Stacy Zimmerman, David Martin, Suporn Sukpraprut-Braaten

**Affiliations:** 1 Internal Medicine, Unity Health, Searcy, USA; 2 Dermatology, Sills Dermatology, Jonesboro, USA; 3 Graduate Medical Education, Unity Health, Searcy, USA; 4 Graduate Medical Education, Kansas City University, Kansas City, USA

**Keywords:** cutaneous symptoms, skin changes, covid and skin, sars-cov-2, covid-19

## Abstract

Individuals infected with SARS-CoV-2 have been found to develop a variety of cutaneous symptoms. This study sought to describe varying cutaneous manifestations of COVID-19 in individuals presenting to an inpatient healthcare facility. We screened individuals who presented with COVID-19 for skin changes throughout the illness and administered a survey regarding demographics, medical history, and their cutaneous findings. Three individuals reported varying skin findings including wheals, petechiae, ecchymosis, and papules. One individual reported a worsening skin condition, psoriasis, as well as a new skin condition, seborrheic dermatitis. In conclusion, cutaneous manifestations of patients suffering from COVID-19 are wide-ranging and worsening skin conditions amongst these patients should be further investigated.

## Introduction

Over the past two years, the world has experienced a rapidly evolving healthcare crisis due to the coronavirus disease 2019 (COVID-19) pandemic. The disease course is highly variable, ranging from asymptomatic carriers to pneumonia with significant complications [1]. In addition to this, there have been a number of cutaneous manifestations associated with the disease that are equally wide-ranging [2-4]. Based on a previous literature review, the prevalence rate for skin changes during COVID-19 infection is between 4.9 and 20.4% [2]. One of the more prominent dermatological diagnoses is pernio, also known as chilblains [2,3]. Chilblains (pernio) or pseudo-chilblains is characterized as violaceous plaques located on the fingers and toes resembling frostbite, without significant exposure to cold and most often found in younger cases of COVID-19 [5,6]. Nevertheless, other presentations have also been recorded including but not limited to maculopapular rashes, purpura, erythema multiforme, urticarial rashes, and vesicular rashes [7].

An individual’s progression of COVID-19 severity may be related to the appearance of specific skin findings [3]. For example, the appearance of retiform purpura has a high association with acute respiratory distress syndrome and thrombotic events [3]. On the other end of the spectrum, most individuals with urticarial or macular erythema experience no complications [3]. Treatment options for these lesions have primarily been based on what was previously known about similar lesions presenting without COVID-19 [8]. Although, it has been hypothesized that treating some of these lesions, such as urticarial rashes may also improve the survival rate of overall COVID-19 [7].

The pathological mechanism of cutaneous manifestations of COVID-19 has not been well studied. However, based on current literature, it is known that the SARS CoV-2 initially infects humans through the binding of spike proteins with the angiotensin-converting enzyme 2 (ACE-2) receptor [9]. The infection then triggers a cascade of local and systemic inflammatory signals, causing widespread damage to several organ systems, ranging from the respiratory to nervous systems [9,10]. Furthermore, a review of a gene expression database has shown that ACE-2 gene expression is higher in human skin keratinocytes and basal cells than in lung tissue [11,12]. This results in a higher number of the ACE-2 receptors, the SARS-CoV2 entry site, in these tissues [9]. Since SARS-CoV2 is most widely considered to be transmitted through aerosol or droplets, the skin is more likely a place for further dissemination of the virus during active infection [13]. Additionally, the pathogenesis of many skin conditions, including psoriasis, atopic dermatitis, and seborrheic dermatitis, is based on sustained inflammation [14-16].

In addition to skin manifestations of COVID-19, the dermatological community warrants further investigation of the effects of COVID-19 on previously diagnosed skin conditions [17,18]. For example, there have been reports of worsening psoriasis with the onset of COVID-19, unexplained by newly prescribed medications or medication withdrawal [17]. One patient who was previously diagnosed with psoriasis, presented with COVID-19, had no medication withdrawal or added new medicines, was found to have worsening psoriatic lesions that improved with the resolution of the disease [17]. Aside from the previously mentioned case, other more extensive studies found evidence of worsening skin conditions during the COVID-19 pandemic [19]. However, there needs to be further examination of the etiology of these changes in the future [18]. To better understand the treatment and prognostic factors of cutaneous manifestations of COVID-19 and the management of current skin disease, we must continue to document these cases as they present to us in the inpatient and outpatient settings. This study presents four cases of COVID-19 who also reported cutaneous manifestations of the disease and a case of worsening prior dermatological diagnoses.

## Case presentation

In this study, there were four individuals who tested positive for COVID-19 by polymerase chain reaction (PCR) who also reported skin changes since symptom onset. They were all seen and interviewed in a single hospital’s emergency department or inpatient setting. These patients were first screened for skin changes after testing by PCR had confirmed these patients to be positive for COVID-19. After consenting to be part of this study, the patients were then asked to complete a brief physician-guided questionnaire that included basic demographics, medical history, history of their COVID-19 illness, and a subjective report of their skin findings. This was then followed by an objective examination of the skin findings by a physician while obtaining photographs of the lesions. Consent was obtained whereby the subjects were guaranteed the right to withdraw from the study, protect their identity, and remove any personal identifiers within the photographs taken. The patients’ demographics, social history, COVID-19-related information, medical history, and cutaneous symptoms are summarized in Tables 1-4.

**Table 1:** Demographics and Social History.

Demographics and Social History	Case 1	Case 2	Case 3	Case 4
Age (years)	51	52	44	47
Gender	Male	Female	Male	Female
Weight (pound)	360	429	177	233
Height (inch)	72	67	72	62
Body Mass Index (pound/inch^2^)	49	67	24	43
Smoking Status	No	No	No	Yes, 13 pack-year
Birth Control	N/A	No	N/A	No
New Medications	No	No	No	Ibuprofen previous night prior to admission

**Table 2:** COVID-19-Related Questions. PCR: polymerase chain reaction

	Case 1	Case 2	Case 3	Case 4
Initial Encounter				
Setting of Interview	Emergency Department	Inpatient	Inpatient	Emergency Department
Disposition	Inpatient Admission	Inpatient Admission	Inpatient Admission	Discharged
Hospital Length of Stay	3 days	2 days	3 days	0 Days
COVID-19-Related Questions				
COVID-19 PCR Testing Result	Positive	Positive	Positive	Positive
Prior COVID-19 Diagnosis	No	No	No	No
Immunization Vaccination Information				
Immunization Vaccination	No	Yes	No	Yes
Immunization manufacturer	N/A	Moderna (Spikevax)	N/A	Janssen COVID-19 Vaccine
Immunization dose	None	First dose in early 2021	None	First dose in June 2021
COVID-19 related Symptoms				
Fever	Yes	No	Yes	Yes
Myalgia	Yes	Yes	No	Yes
Fatigue	Yes	Yes	Yes	Yes
Shortness of Breath	Yes	No	No	Yes
Other	Chills	Dizziness	Nausea/Vomiting	Eye redness, dizziness

**Table 3:** Medical History. *CVA/TIA = cerebrovascular disease/transient ischemic attack ** DVT = deep vein thrombosis

Medical History[SB1]	Case 1	Case 2	Case 3	Case 4
Cancer	No	No	No	No
Skin Disorder	No	No	Yes, Psoriasis	No
Hypertension	Yes	Yes	Yes	Yes
Myocardial Infarction	No	No	No	No
Renal Failure	No	No	No	No
Diabetes	Yes	No	Yes	No
Prior CVA/TIA^*^	No	No	No	No
Prior DVT^**^	No	No	No	No
Prior Pulmonary Embolism	No	No	No	No
Immunocompromised	No	No	No	No
Vasculitis	No	No	No	No
Autoimmune Disorder	No	No	No	No
Immune Modulating Drugs	No	No	No	No

**Table 4:** Cutaneous Symptoms.

COVID-19 Cutaneous Symptoms	Case 1	Case 2	Case 3	Case 4
Self-described changes immediately surrounding COVID-19 diagnosis	Red dots on both ankles first noticed by his wife.	Left leg bruise followed by skin coming off. Denied any trauma to the area. Looks like left shoulder prior to skin coming off. Pimple like spots on the chest. Another area of skin sloughing in left axilla that hadn’t been noticed previously.	Dry, flaky, red skin breakout on the forehead and cheeks. Burns but doesn’t itch.	Redness and burning along the forehead, scalp, ears, neck. Took ibuprofen last night, rash was then worse in the morning.
What skin conditions suffered from previously worsened since COVID-19 diagnosis?	No	No	Yes, Psoriasis	No

Case 1

**Figure 1:**
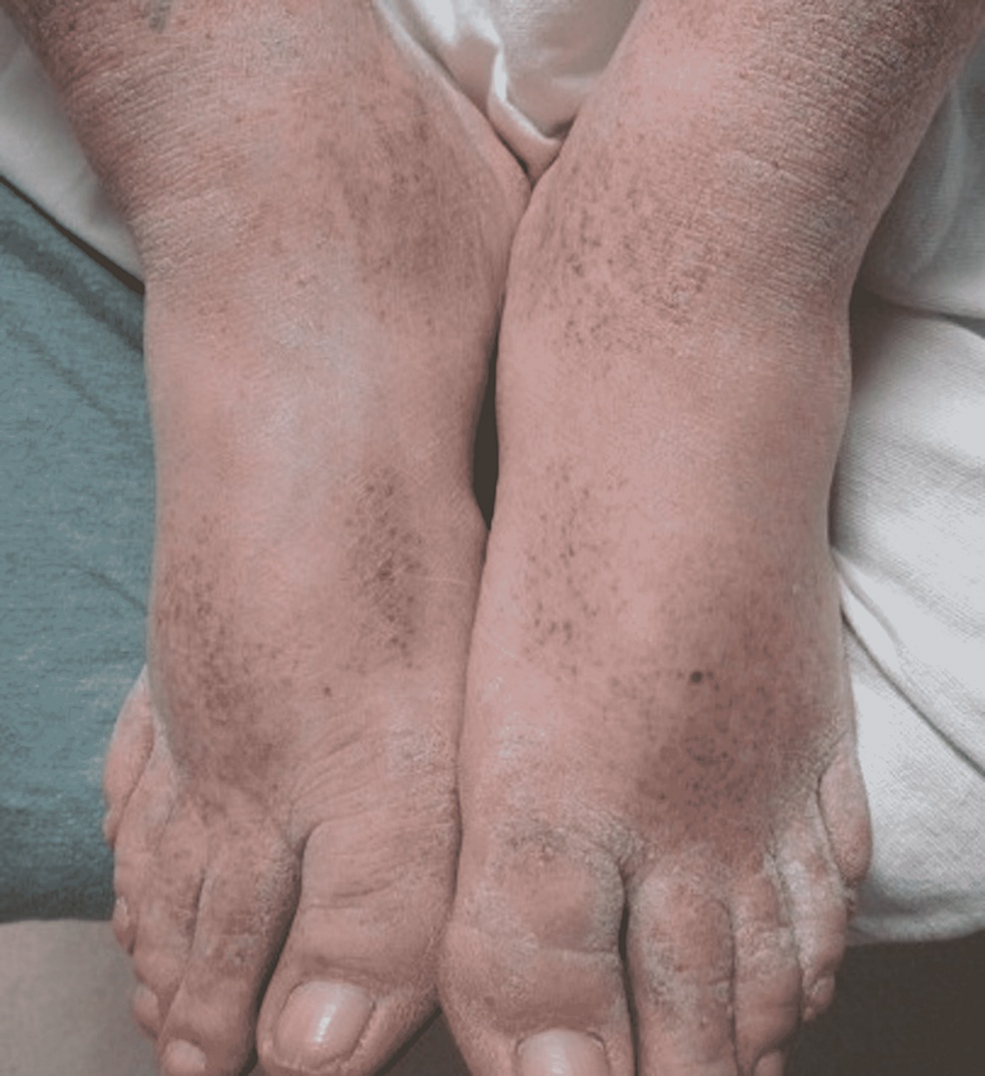
Petechiae Along the Dorsal Surfaces of the Feet and Anterior Ankles.

**Figure 2:**
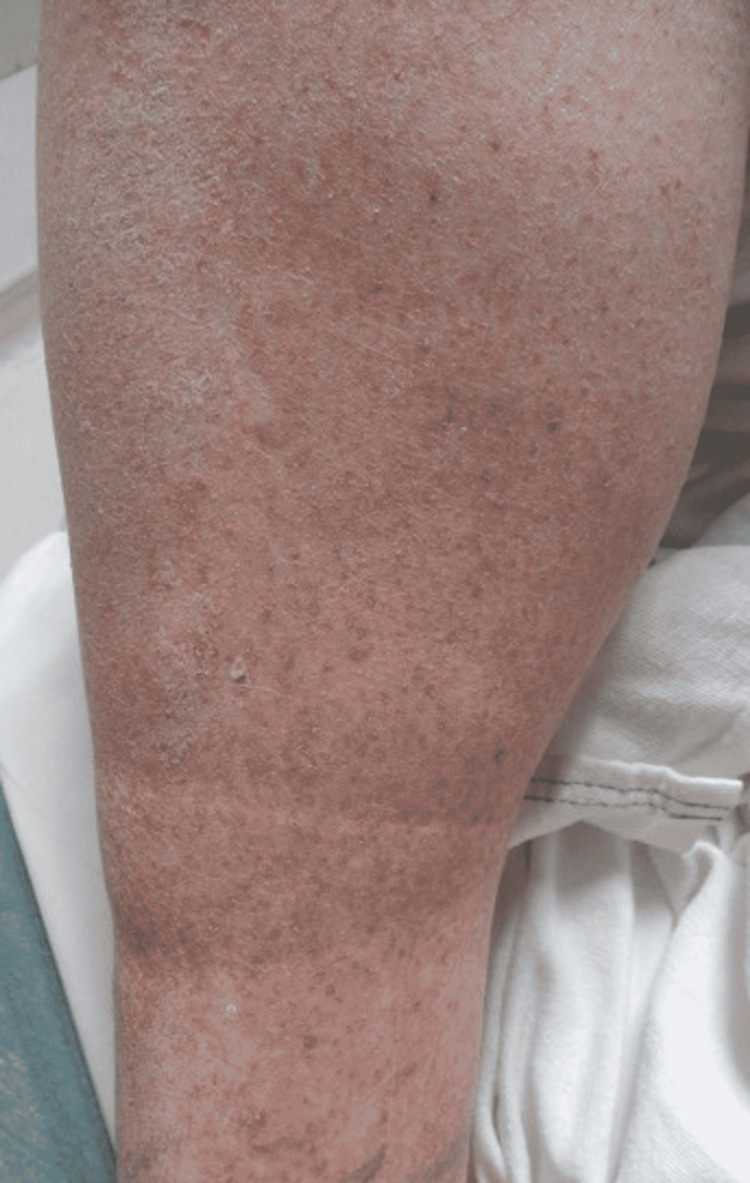
Petechiae Along the Right Anterior Pretibial Surface.

**Figure 3:**
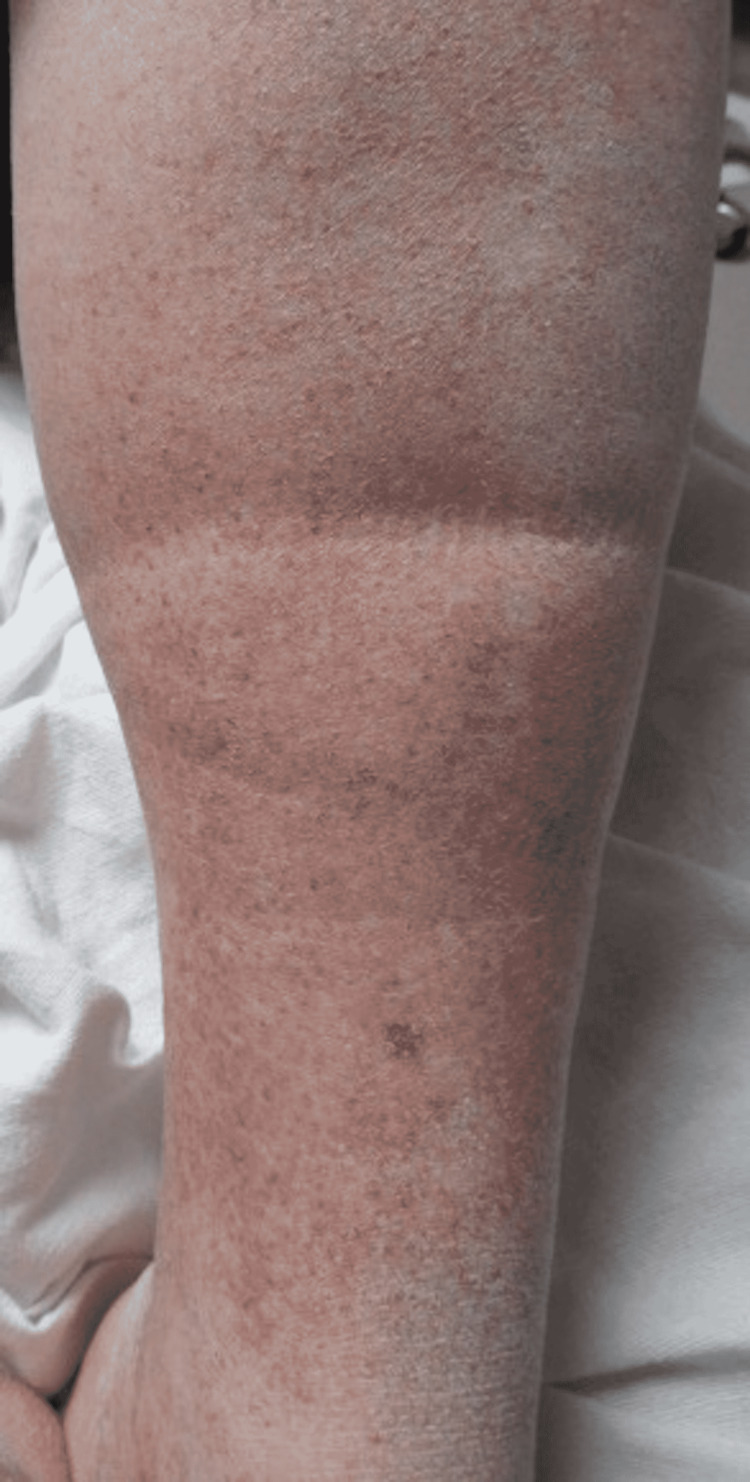
Petechiae Along the Left Anterior Pretibial Surface.

A 51-year-old male presented to the Emergency Department due to shortness of breath, requiring oxygen supplementation. The patient was subsequently diagnosed with COVID-19 after testing positive for the disease by PCR. He had no previously diagnosed skin conditions to his knowledge. Upon examination, he had a petechial rash along the bilateral feet (Figure 1) and lower extremities up to the knee (Figures 2-3). Of note, he had not noticed the lesions himself until his wife mentioned them to him. Due to staffing shortages, the patient stayed in the Emergency Department overnight while awaiting an inpatient room. The images of the reported skin changes were taken the following day. The patient reported significant improvement in the lesions since presentation, which was confirmed by the physician’s physical exam. After presentation to the ED, non-invasive ventilation was initiated due to hypercapnia. Over the course of one night, the patient’s respiratory symptoms improved and the patient then continued supplemental oxygen with a nasal cannula until shortly before discharge.

Case 2

**Figure 4:**
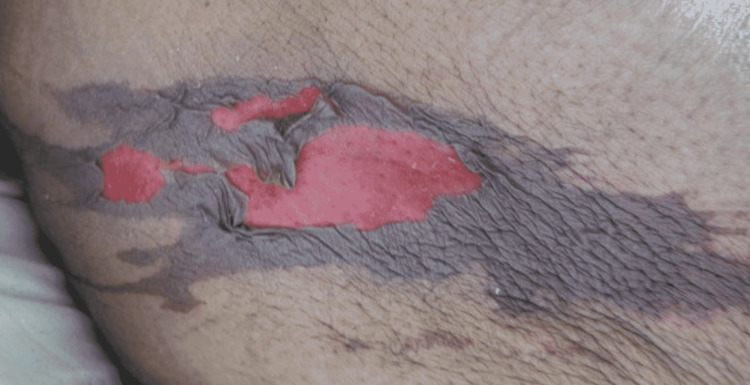
Well-Defined Ecchymosis With Centrally Denuded Epidermis on the Left Lateral Thigh.

**Figure 5:**
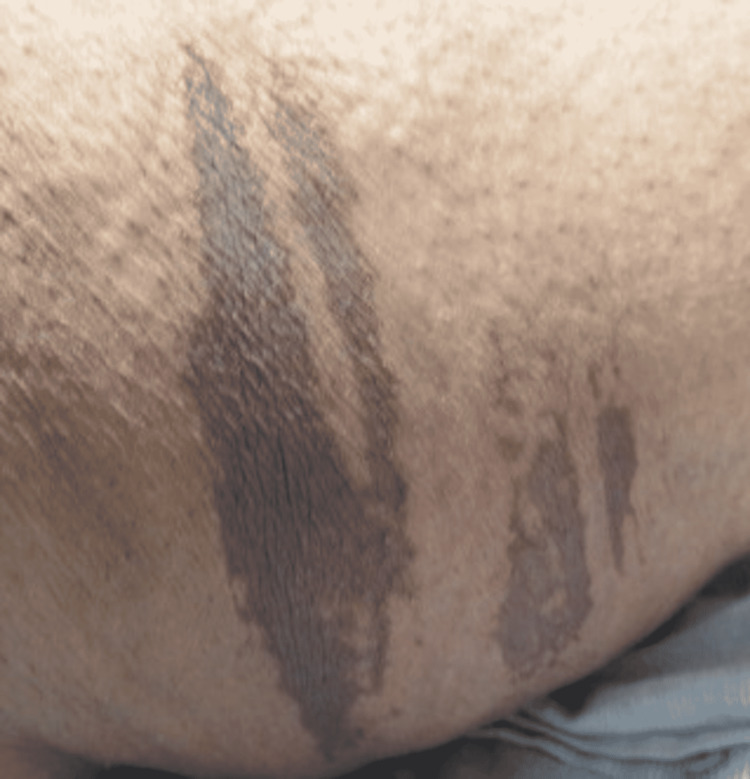
Well-Defined Ecchymosis on the Left Shoulder.

**Figure 6:**
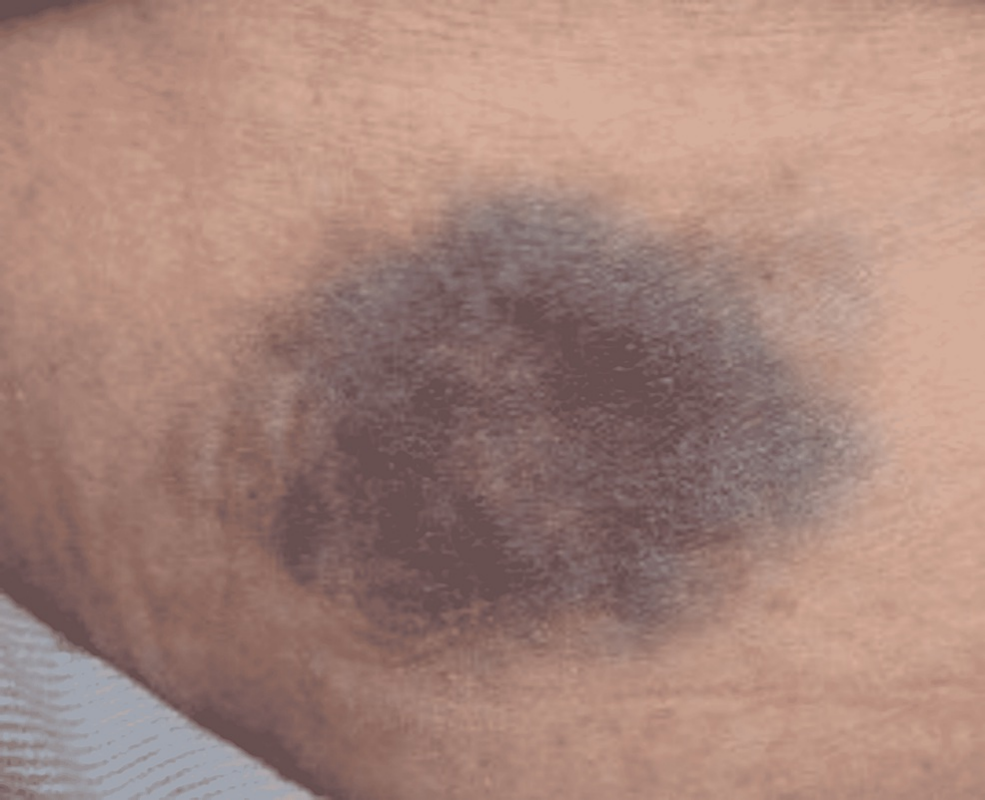
Violaceous Purpuric Patch With Hazy Borders on the Left Axilla.

**Figure 7:**
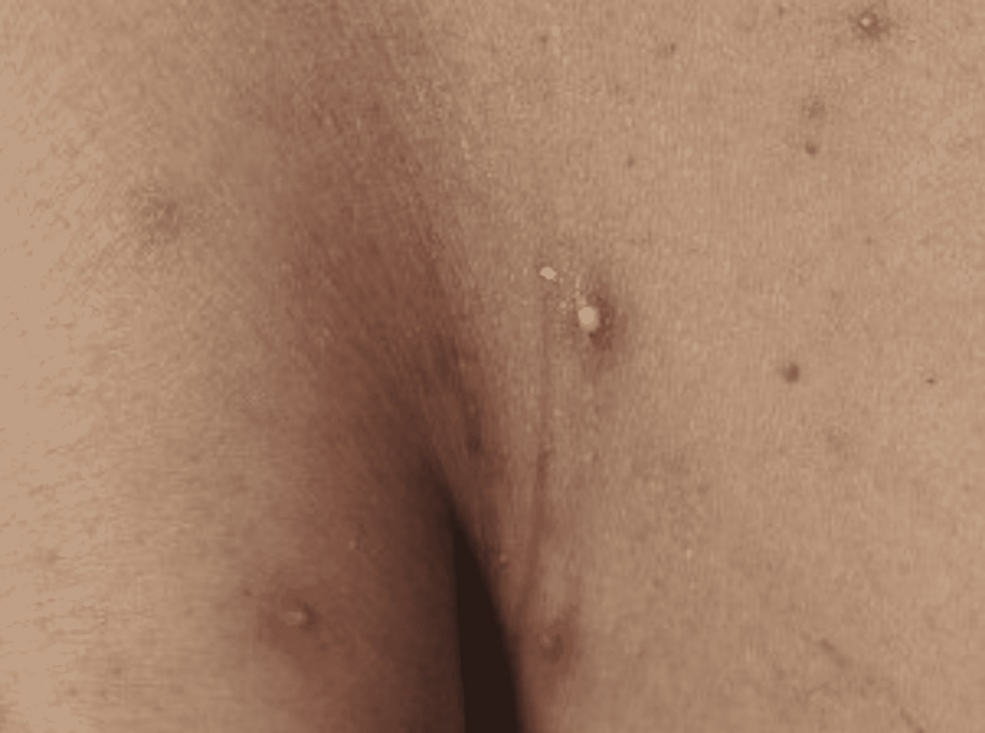
Multiple Pustules and Erythema-Tous Papules on the Chest.

A 52-year-old female presented to the Emergency Department (ED) due to overall weakness and dizziness. She stated that she had not been able to function as well over the previous two days. The patient tested positive for COVID-19 by PCR. She did not require oxygen supplementation but did require supportive care. At the time of the interview, the patient had been admitted for inpatient care. The patient stated that she had two different lesions, one along the left thigh that had arisen over the past day. It first appeared as a purple spot that then darkened, and “bubbled,” at which point the top layer of skin came (Figure 4). There was another lesion on the left shoulder that had the appearance of the first lesion before the skin “sloughing” (Figure 5). There was an additional ecchymotic lesion that had the appearance of the other two lesions in the first stage of their evolution on the left axilla (Figure 6). She denied having any recent trauma or previously diagnosed skin conditions. In addition to the lesions above she also stated that there were small spots on her chest, appearing vesicular, that had arisen after the onset of COVID-19 symptoms (Figure 7). No additional treatment was administered for the patient’s skin lesions.

Case 3

**Figure 8:**
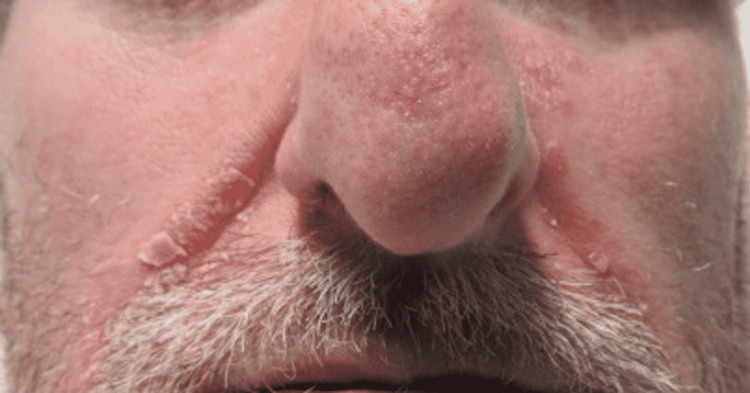
Erythematous Patches With Greasy Scale Along the Melolabial Folds.

**Figure 9:**
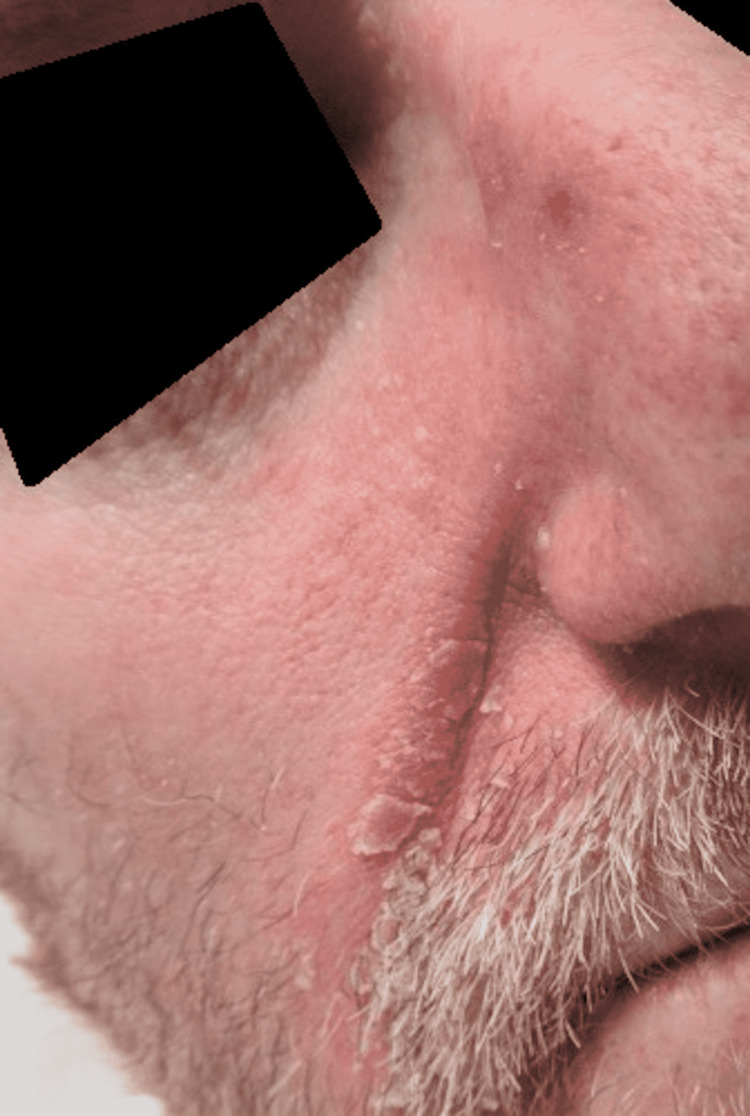
Erythematous Patch With Greasy Scale Along the Melolabial Fold and Medial Cheek.

**Figure 10:**
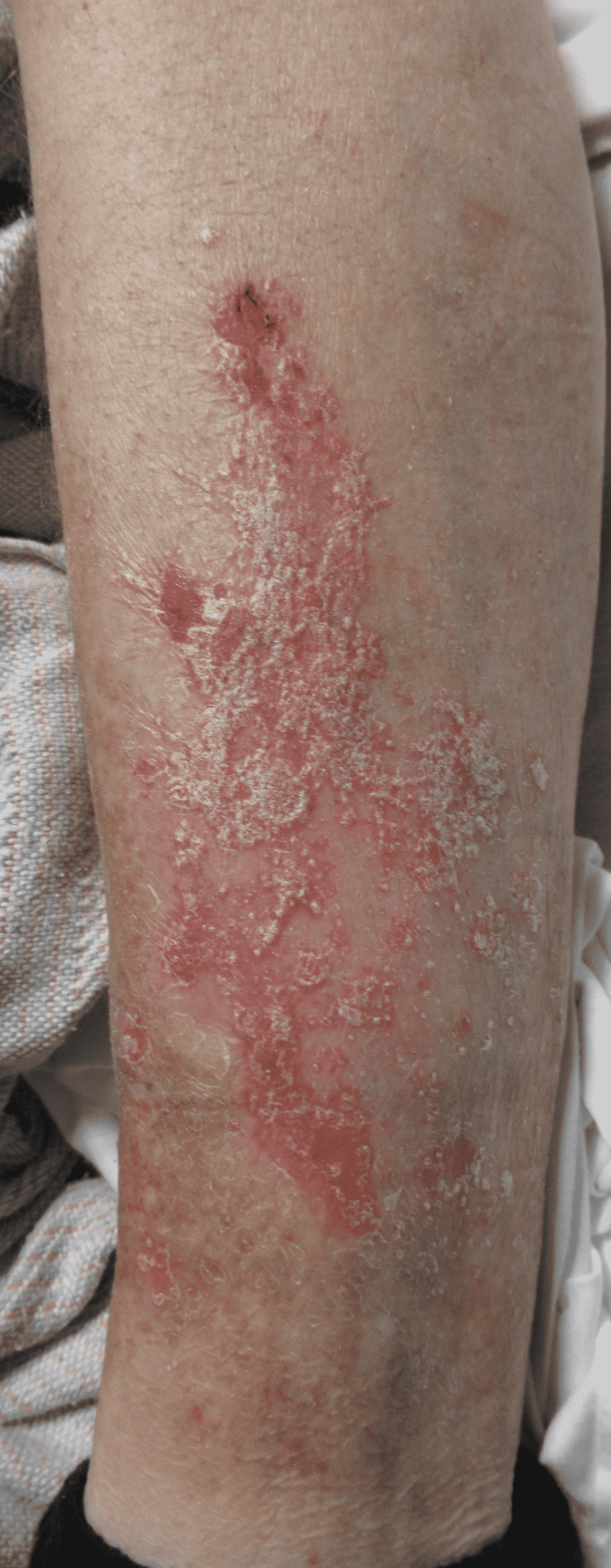
Large Erythematous, Pruritic, Plaque With Silvery Scale Along the Left Pretibial Area.

**Figure 11:**
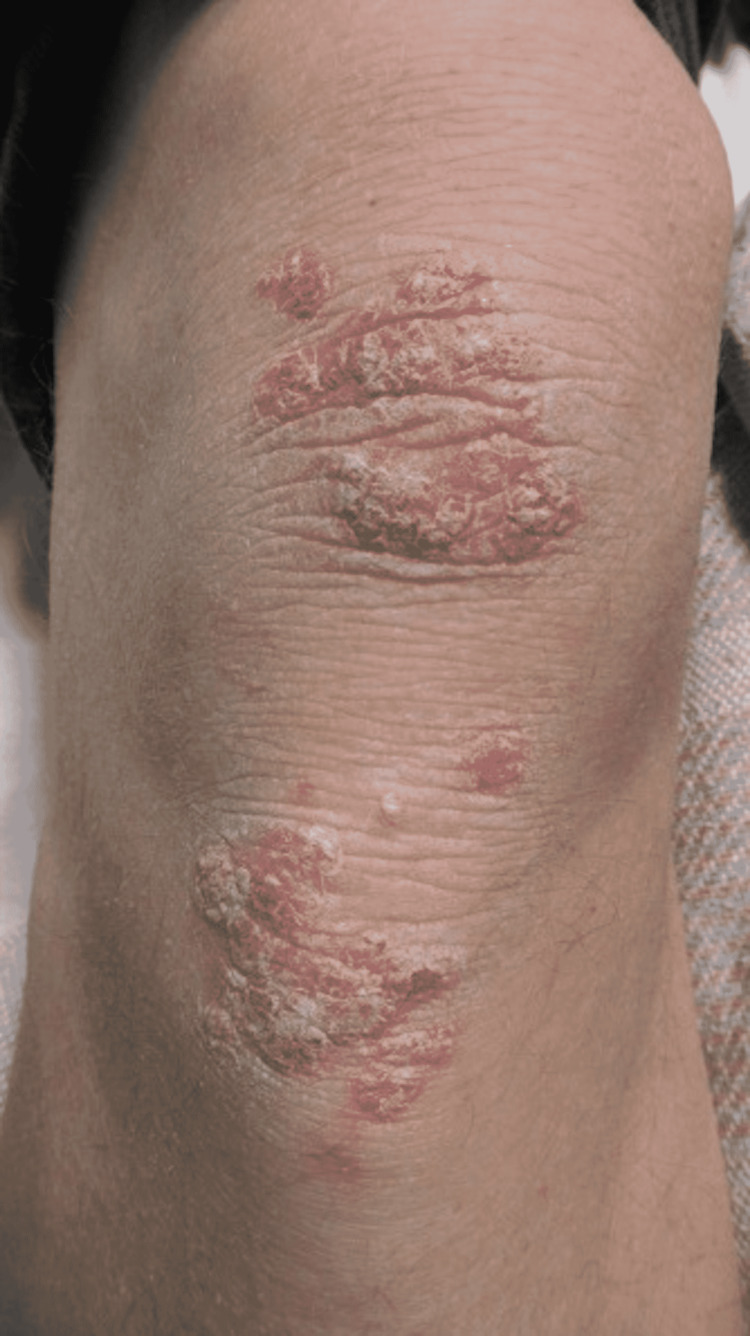
Erythematous, Pruritic Plaques With Silvery Scale Along the Right Knee.

A 44-year-old male presented to the Emergency Department due to weakness, fatigue, intractable vomiting, and anorexia. For over a week, he had been experiencing these symptoms but had worsened over the last three days, which was why he sought medical care. The patient tested positive for COVID-19 by PCR. With normal saline fluid resuscitation and treatment with antiemetics, his symptoms improved over two days. He was found to have worsening erythematous plaques with greasy scales along the forehead, and bilateral malar regions that had appeared in the past week with the onset of his illness (Figures 8-9). He denied itchiness but stated that the lesions burned. In addition to these lesions, he had erythematous plaques with silver scales along the bilateral elbows and knees and the left pretibial region (Figures 10-11). Although he stated that he had never been diagnosed with a skin condition by a physician, these lesions appeared to be consistent with plaque psoriasis. According to the patient, these lesions had worsened significantly since the onset of COVID-19 symptoms. He was prescribed betamethasone diproprionate cream, and over the course of the next two days, the treatment improved his lesions.

Case 4

**Figure 12:**
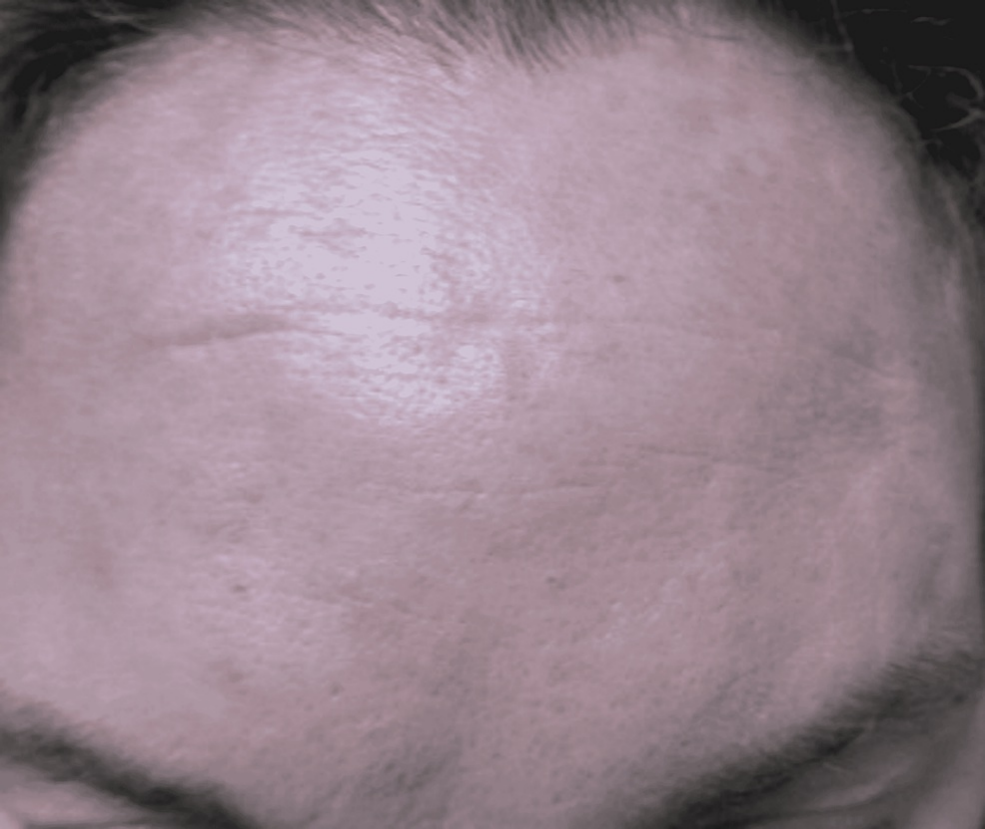
Multiple Pruritic Wheals.

A 47-year-old female presented to the emergency department due to fever, fatigue, myalgia, shortness of breath, and overall concern for COVID-19. The patient tested positive for COVID-19 by PCR. On exam, she was found to have erythematous patches along the forehead and right post-auricular region (Figure 12). She stated that the lesions had developed since the onset of COVID-19 symptoms and complained of a burning sensation. Of note, she did admit to ingesting ibuprofen the night before presentation. The lesions were present before ingesting ibuprofen but had worsened overnight. She had never experienced a reaction to the drug in the past. She was not hypoxic as judged by an oxygen saturation greater than 92% and thus had no oxygen supplementation. She was considered to be stable and discharged home from the ED.

## Discussion

This study reemphasizes the array of COVID-19-induced cutaneous manifestations that can be seen in a healthcare setting. Earlier studies have examined the number of different presentations of COVID-19 on the skin that have arisen since the onset of the pandemic [3,4,7,20]. The cutaneous manifestations that have been reported in this study are mainly in line with other large-scale studies regarding cutaneous manifestations of COVID-19, including urticaria, petechia, purpura, and vesicular rashes [3,4,7,20]. Most subjects infected with COVID-19 who reported skin changes experienced mild disease overall and experienced no further complications [3]. This study enforces these findings as none of the subjects listed experienced serious COVID-19 complications or required intubation with ventilator support. Only one of the subjects in this study needed supplemental oxygen. Over three days the subject improved and was discharged home without further complications. This patient did suffer from a vascular type rash, petechia, which has been associated with worse outcomes in another study [2]. Subjectively, three out of four patients rated their illness as at least 8 in COVID-19 symptoms severity. All patients reported fatigue, and three patients reported either myalgia or fever. Only two of the patients reported having had at least one dose of COVID-19 vaccination, regardless of manufacturer. All four patients reported a history of hypertension, and two reported a history of diabetes. 

Demographically, the subjects in this study are similar to those diagnosed by PCR in another more extensive literature review, including in age, which had a mean of 48 years, compared to 44 years reported by Freeman et al. and 49 years reported by Zhao et al [3,4]. Similar to a majority of other patients reporting skin findings, patients in this study all experienced cutaneous manifestations following symptoms of COVID-19, rather than skin findings being the first indication of COVID-19 [3]. Also of note is the vaccination status of the patients in this study. Two were vaccinated against COVID-19, one of the two fully vaccinated, and both received different vaccination types. There have been reactions to vaccination for COVID-19 manifested on the skin and the appearance of autoimmune disease [21]. Notably, some of the reaction types are similar to cutaneous changes seen during active COVID-19, which raises questions regarding pathophysiological mechanisms, including molecular mimicry through viral proteins [21]. 

In addition to these more common presentations, we did note the appearance of seborrheic dermatitis that worsened over the patient’s course of COVID-19 illness. The patient referenced in Case 3 had never experienced lesions like this in the past, and their appearance occurred after the development of COVID-19 symptoms. A case similar to this, with new-onset seborrheic dermatitis during COVID-19 has been recorded in a case study previously [22]. There were some differences in this case, notably, the condition of the subject was more critical than the subject reported in this case [22]. Nevertheless, the similarities are consistent in the sense of developing a new skin condition after the onset of COVID-19 [22]. The clinician must be aware of the concomitant development of skin conditions, such as seborrheic dermatitis with COVID-19, so that they can be treated appropriately and further monitored for further development of the condition after recovery.

There have also been instances of worsening pre-existing conditions, such as psoriasis, with the development of COVID-19 symptoms [17,18]. One case study reported psoriatic plaques present for an extended period. However, the lesions had acutely worsened with the development of COVID-19. Also noteworthy, the patient has never undergone treatment for the plaques or was taking any medications. Another case report suggests the patient’s psoriasis worsened due to hydroxychloroquine used to treat the patient’s COVID-19 [23]. The patient presented in this study supports the findings of Ozaras et al., considering the patient was abstinent from medications and had worsening psoriatic lesions in the setting of COVID-19 [17].

There are very few recommendations from the American Academy of Dermatology (AAD) regarding COVID-19 and cutaneous manifestations [24]. Clinical guidance resources published by the AAD ranged from personal protective equipment (PPE) and medical supplies, office management, and management of immune-modulating therapeutics [25]. The most recent 'Statement on Dermatologic Manifestations of COVID-19' by the AAD urges evaluation of a single type of lesion, pernio or chilblains, and does not elaborate on other lesions seen during the infection [26]. Even though the AAD has not been involved extensively in creating significant guidelines regarding skin lesions during COVID-19, there have been tremendous efforts to log the vast array of lesions being seen globally through a central database created by the organization [3]. With this information, the medical community has attempted to better understand the prognostic value of specific skin lesions during COVID-19 [3,27]. 

In addition to new lesions, it will be essential to monitor for worsening skin conditions diagnosed before COVID-19 infection, such as the case of worsening psoriasis [17]. Treatment recommendations regarding COVID-19 and cutaneous symptoms are often based upon targeted therapeutic approaches [8]. It should also be noted that some lesions may require treatment with corticosteroids, which is recommended in some cases of COVID-19 [8]. A prophylactic approach to COVID-19 might also include supplementation with Vitamin D, which has been shown to reduce the severity of COVID-19 as well as many skin conditions [28,29].

## Conclusions

This study aims to further build upon our current understanding of COVID-19 and the presentation of the disease through the skin. In addition to more common cutaneous manifestations of COVID-19, new-onset and worsening of other skin conditions during the infection are described in this series. The study also builds awareness of the effects of COVID-19 on patients who have previously diagnosed skin conditions and how the clinician might manage worsening conditions during the illness and after recovery. The clinician should also be aware of the skin conditions that are more likely associated with mild courses of COVID-19, such as urticaria, and those more likely to be associated with more severe disease, such as petechia or retiform purpura. Awareness of clinical manifestations of COVID-19 is vital for the clinician to reassure patients presenting to them regarding the prognosis of the disease and offer supportive care if the lesions affect the quality of life. There were limitations to this study, including the patient population, being only those who presented to the hospital setting. There is also the possibility that there is recall bias or limitation in recall amongst the subjects. Many of the COVID-19 cases with cutaneous manifestations are mild and only present in an outpatient clinic. In addition, there may have been some reporter bias as the patients with more significant and bothersome skin lesions made the physician aware that they had appeared with the onset of COVID-19.
